# Supplementary material for: Dynamic construction of refractive index-dependent vibrations using surface plasmon-phonon polaritons
Source: Nat Commun. 2023 Nov 11;14:7316. doi: 10.1038/s41467-023-43127-z (PMC10640644; doi:10.1038/s41467-023-43127-z)
Supplement: Supplementary file 1 — Supplementary Information [file 41467_2023_43127_MOESM1_ESM.pdf]

Supplementary Information for

Dynamic Construction of Refractive Index-Dependent Vibrations Using Surface Plasmon-Phonon Polaritons

Hong Zhou, Zhihao Ren, Dongxiao Li, Cheng Xu, Xiaojing Mu, Chengkuo Lee**

Table of Contents

Supplementary Note 1. More discussion on stacked nanoantennas-----------------------------------2

Supplementary Note 2. The radiating oscillator model---9

A. Modeling plasmon-phonon coupling---9

B. Modeling the phonon polariton branches--11

Supplementary Note 3. More discussion on polariton-molecule interaction---------------------13

Supplementary Note 4. ALS algorithm for multispectral baseline fitting ------------------------16

Supplementary Note 5. DNN model training and validation---19

A. DNN model for SP-PhP platform---19

B. DNN model for nanorod platform--21

Supplementary Note 6. Kramers–Kronig relations --23

Supplementary Note 7. Complex Refractive index calculation ---------------------------------------25

Supplementary Figure 13. Thickness-dependent transition of plasmon-phonon coupling----27

Supplementary Figure 14. BSA thickness characterization---28

Supplementary Figure 15. Noise analysis of the experimental setup--------------------------------29

Supplementary Figure 16. Environmental disturbances--30

Supplementary Figure 17. Nanofabrication processes--31

Supplementary Table 2. Mode splitting of SP-PhP platform---32

Supplementary Table 3. Comparison of infrared antenna-based biosensors ---------------------33

Supplementary References---34

Supplementary Note 1: More discussion on stacked nanoantennas

Supplementary Figure 1a shows the schematic of the parallel nanoantennas. When the external magnetic **H** and electric **E** field is applied parallel to the boundary of the antenna, the electric currents in the parallel antennas along the *x-axis* are excited. One case is that the current intensities are different in the two antennas because of the nonbalanced trapezoidal structure. The different current intensities will lead to the net current I_x being nonzero. Therefore, a displacement current is excited between the antennas and a virtual current loop will be formed along a contour [$\mathbf{P}_1$, $\mathbf{P}_4$, $\mathbf{P}_2$, $\mathbf{P}_3$]. According to Faraday's law, a magnetic moment is induced. Notably, the direction of the induced magnetic field $\mathbf{H}_{\text{ind}}$ is opposite to that of the external magnetic field **H**. Supplementary Figure 1a shows the broadband resonance of the parallel nanoantennas when the two resonant modes are in proximity to each other. The bandwidth of the parallel nanoantennas is 1.5 times wider than that of the nanorod. Supplementary Figure 1c-f shows the distribution of the z-axis electric and magnetic field components along the 2D xz plane. The bonding mode presents a displacement current with identical flow directions in the two parallel antennas, under the driving of a time-varying electromagnetic field. Conversely, the antibonding mode displays opposite current directions between the parallel antennas, forming a virtual current loop due to the unequal current intensities.

Supplementary Figure 2a shows the plasmonic stacked nanoantenna with out-of-plane coupling. According to the sectional view of E-field distribution calculated by simulations, the lightning-rod effect of plasmonic field enhancement is observed on both bonding and antibonding modes, due to the excitation and concentration of plasmons at the edges of nanostructures (Supplementary Figure 2b,c). The length L of the antenna has a direct linear impact on the resonance of the coupled system (Supplementary Figure 2d). Antenna width W and cell periods $P_{\text{x,y}}$ affect the bandwidth of the system (Supplementary Figure 2e-i), and the effects are different. When the period of the antenna is small, multiple plasmonic oscillation modes are generated on the antenna, and the frequency distribution of these modes is relatively wide, which can support a wider bandwidth. For the effect of width, a wider antenna can support more plasmonic oscillation modes, leading to a wider bandwidth. Because a wider antenna provides a larger plasmon volume and more degrees of freedom for plasmonic oscillations. However, if the antenna is too wide, the plasmon density becomes non-uniform, leading to attenuation of the plasmonic antenna performance.

The thickness of each layer of the stacked antenna also affects the stacked nanoantenna behavior (Supplementary Figure 3a). When the thicknesses of mental antennas are equal (t_1=t_3, Supplementary Figure 3b), an increase in the thickness of the spacer enlarges the splitting between the two modes. Because the increase in the spacer thickness reduces the strength of the magnetic response between the two metal antennas. When the spacer thickness t_2 is fixed and $t_1 \neq t_3$, (Supplementary Figure 3c), splitting occurs at the configuration with larger values of t_1 or smaller values of t_3. It implies the sensitivity of the stacked nanoantenna to the direction of incidence of the light. The light incident from the thicker antenna to the thinner antenna is easy to excite the mode splitting. These interesting properties of the stacked nanoantenna can be used in the broadband design, that is, to find the starting point of splitting.

Additionally, there is no significant difference observed in the polarization response between the stacked antennas with stacked nanoantenna and the mono-layer nanorod (Supplementary Figure 4). Both the stacked antenna and the single-layer nanorods exhibit a polarization variation range of 35°, within which the resonance strength of the antenna exceeds half of that observed when the polarization angle is 0 (Supplementary Figure 4c,d).

Regarding the angle of incidence range, the stacked nanoantenna also does not affect the effective range of the antenna’s angle of incidence (Supplementary Figure 5). Both the stacked antenna and the mono-layer nanorod have a 50° variation range, where the resonance strength is greater than half that at normal incidence (Supplementary Figure 5c,d).

In terms of novelty, we capitalize on the partial overlap of the two modes in the stacked antenna when they are in proximity to each other, thereby achieving broadband resonance. This method is effective and has not been reported in previously reported work. Current broadband strategies, such as supercells[1] and multi-pixel designs[2], exploit in-plane space to achieve the goals. Our method adopts an out-of-plane strategy, which efficiently avoids occupying the in-plane space. Consequently, our approach remains fully compatible with all existing methods while offering the potential to significantly enhance the bandwidth of these existing techniques.

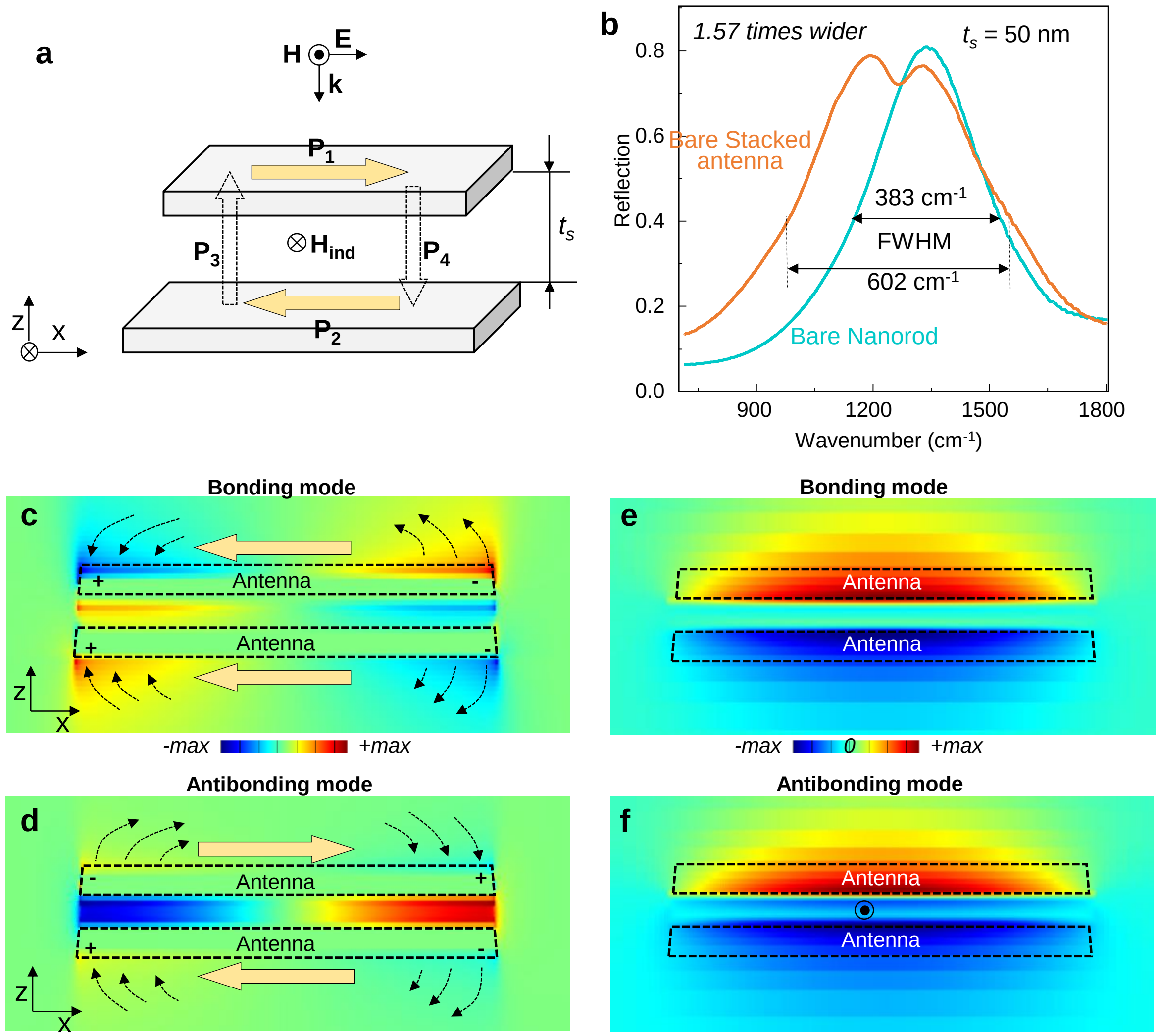

Supplementary Figure 1. Optical response of stacked mental antennas in a parallel plate configuration. a, Schematic of the parallel nanoantennas. Two resonant modes become excited: a lower-frequency bonding mode and a higher-frequency antibonding mode. **b**, Experimental results showing the broadband resonance of the parallel nanoantennas when the two resonant modes are in proximity to each other. **c,d,e,f**, The distribution of the z-axis electric and magnetic field components along the 2D xz plane. (c,e) is known as the antibonding mode, where two antennas have the same direction of (c) the electric field components and (e) the magnetic response between them is weak. (d,f) is known as the bonding mode, where (d) the direction of the electric field is opposite and (f) a significant magnetic response between the parallel antennas is observed.

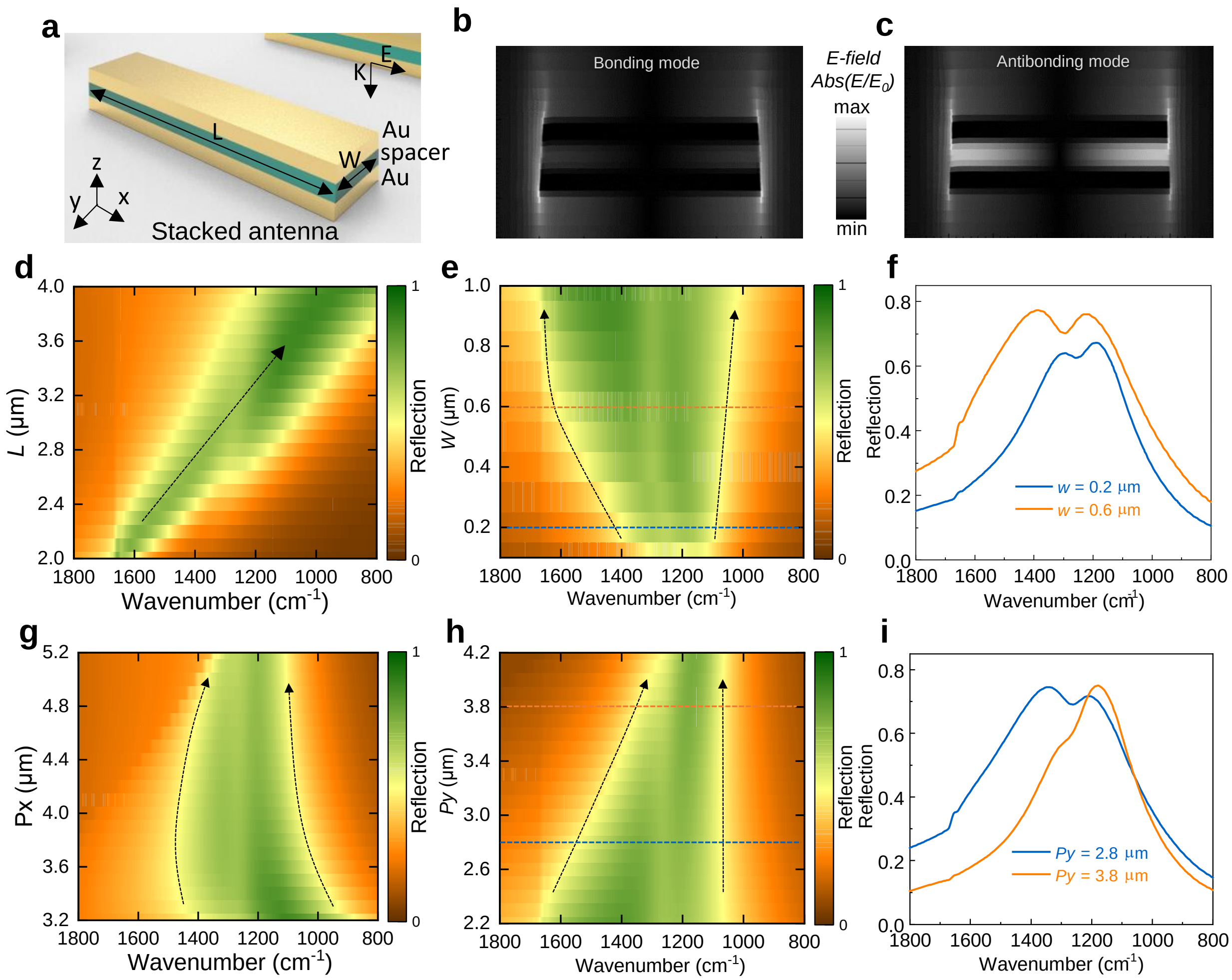

Supplementary Figure 2. Geometry-dependent characteristics of stacked nanoantennas. a, Schematic of the plasmonic stacked nanoantenna. **b,** Sectional view of E-field distribution of bonding mode and **c,** antibonding mode. **d,** Antenna length *L* and **e,** width *W versus* spectrum wavenumber mapping. **f,** Spectra of the device with $W = 0.2$ μm and 0.6 μm. **g,** unit cell period in the x-axis P_x and **h,** y-axis direction *Py versus* spectrum wavenumber mapping. **i,** Spectra of the device with $W = 2.8$ μm and 3.8 μm.

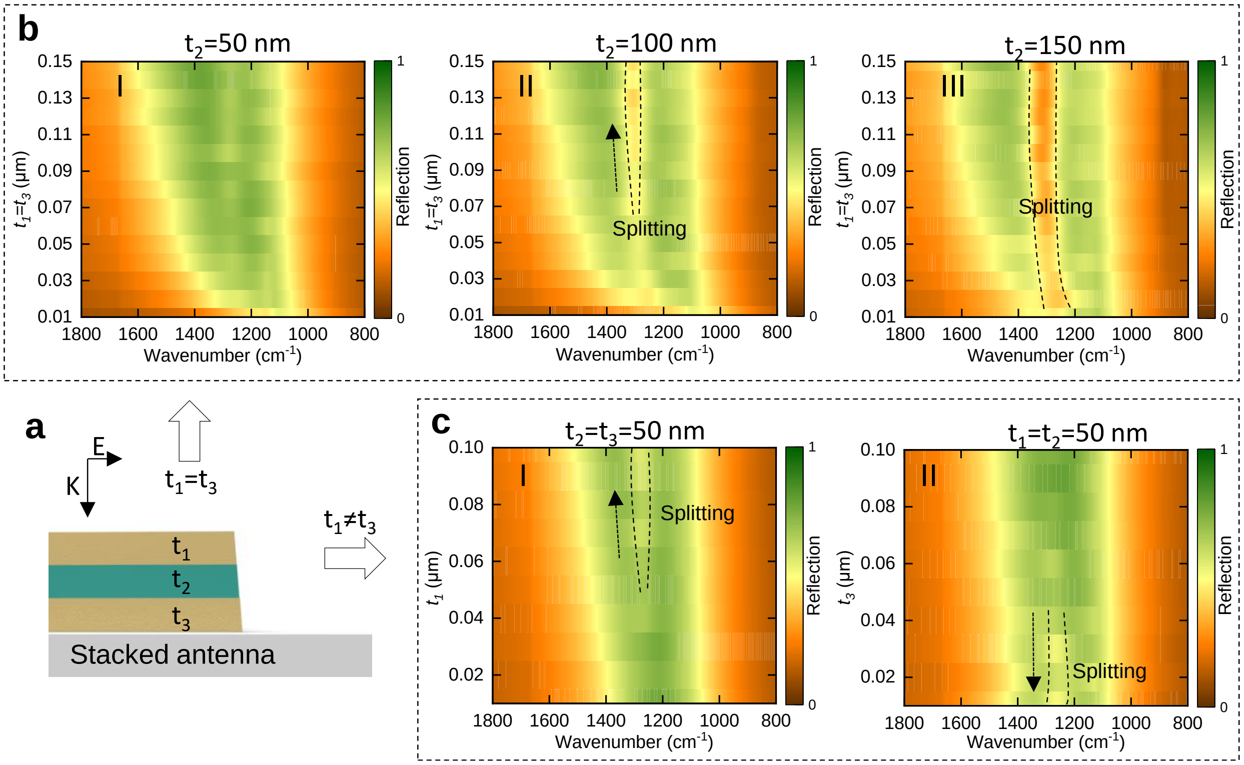

Supplementary Figure 3. Thickness-dependent characteristics of stacked nanoantennas. a, Sectional view of the plasmonic stacked nanoantenna. W= 300 nm. L =3 μm. **b,** Thickness *versus* spectrum wavenumber mapping when the stacked Au antenna thicknesses are equal t_1=t_3 varying from 10 nm to 150 nm and spacer thickness t_2= 50 nm, 100 nm, and 150 nm. **c,** The spectral response mapping when $t_1 \neq t_3$. t_1 or t_3 changes from 10 nm to 100 nm, and t_2 remains unchanged.

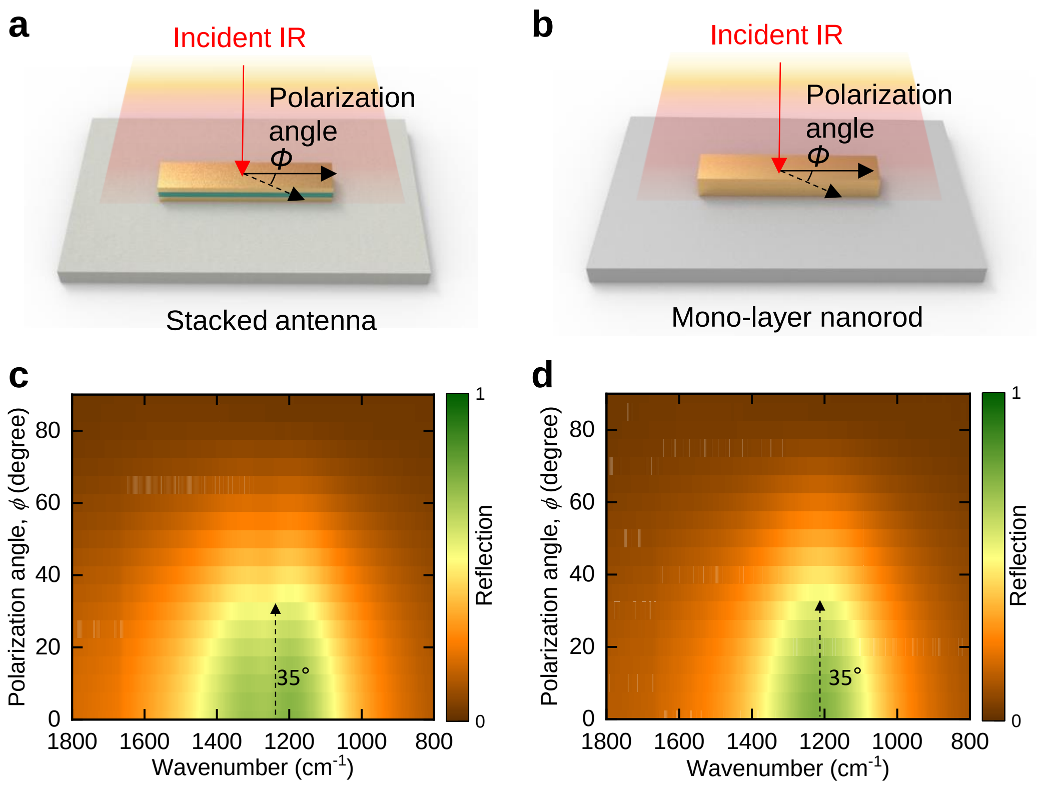

Supplementary Figure 4. Polarization characteristics of stacked nanoantennas. a, Schematic of the stacked antenna and **b,** common mono-layer nanorod with a varying polarization angle. t_1= t_2= t_3 = 50 nm. W= 300 nm. L =3 μm. **c,** Polarization angle versus spectrum wavenumber mapping of the stacked antenna. **d,** The polarization performance of the mon-layer nanorod. Both the stacked antenna and the mon-layer nanorod have a varying range of 35°, in which the resonance intensity is greater than that when the polarization angle is 0.

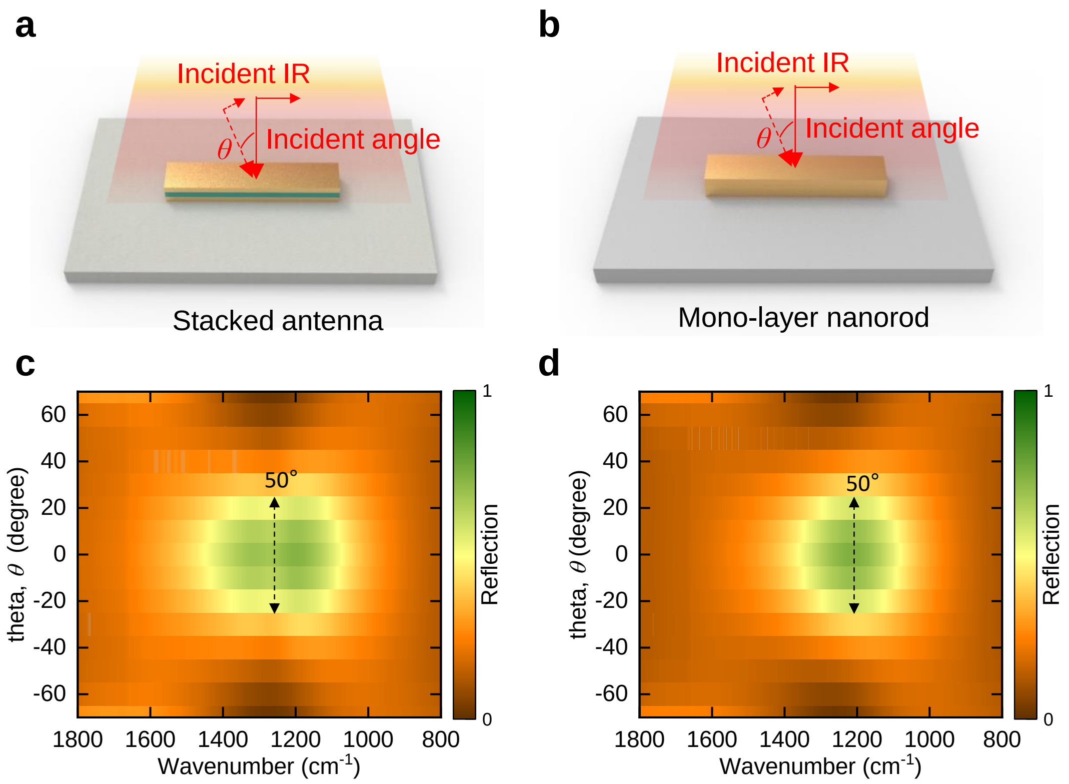

Supplementary Figure 5. Incident range characteristics of stacked nanoantennas. a, Schematic of the stacked antenna and **b,** common mon-layer nanorod with a varying incident angle. $t_1 = t_2 = t_3 = 50$ nm. $W = 300$ nm. $L = 3$ μm. **c,** Incident angle θ versus spectrum wavenumber mapping of the stacked antenna. **d,** The performance of the mon-layer nanorod. Both the stacked antenna and the mon-layer nanorod have a varying range of 50°, in which the resonance intensity is greater than that at normal incidence.

Supplementary Note 2: The radiating oscillator model

A. Modeling plasmon-phonon coupling

Our SP-PhP platform involves the coupling of surface plasmon polaritons and phonon polaritons. To describe this coupling system, we use a classical model comprising two coupled harmonic oscillators that are subject to external forces $f_1(t)$ and $f_2(t)$. The model includes a dark oscillator that represents the purely dissipative mode of the phonon. The model can be expressed as follows:

$$\ddot{p}_1(t)+\gamma_1\dot{p}_1(t)+\omega_1^2 p_1(t)= f_1(t)-\mu\exp(i\varphi)p_2(t)-\kappa_1\exp(i\varphi_1)q(t) \tag{S1}$$

$$\ddot{p}_2(t)+\gamma_2\dot{p}_2(t)+\omega_2^2 p_2(t)= f_2(t)-\mu\exp(i\varphi)p_1(t)-\kappa_2\exp(i\varphi_2)q(t) \tag{S2}$$

$$\ddot{q}(t)+\gamma_\mathrm{m}\dot{q}(t)+\omega_\mathrm{m}^2 q(t)=-\kappa_1\exp(i\varphi_1)p_1(t)-\kappa_2\exp(i\varphi_2)p_2(t) \tag{S3}$$

The excitation, damping factor, and resonance frequency of the bright-mode resonators 1 and 2 are represented by $p_{1,2}$, $\gamma_{1,2}$, and $\omega_{1,2}$, respectively. The two resonators are linearly coupled with each other through a coupling strength $\mu\cdot\exp(i\varphi)$, where the phase shift φ between the two resonators is induced by the retardation effect. The dissipative phonon oscillator, which has a resonance frequency of ω_m and damping factor γ_m, is excited by $q(t)$, and its coupling strength with the plasmonic oscillator is represented by $\kappa_{1,2}$. We make the assumption that $f_1(t)= f_2(t)= f(t)$, $\exp(i\varphi_1)= c_1$ and $\exp(i\varphi_2)= c_2$. In this work, the phonon is coupled with the bonding mode of nanoantennas, so κ1 is equal to 0. Under steady-state conditions, $p_1(t)$, $p_2(t)$, $q(t)$ and $f(t)$ can be expressed as:

$$\begin{bmatrix} p_1(t) \\ p_2(t) \\ q(t) \\ f(t) \end{bmatrix} = \exp(-i\omega t)\begin{bmatrix} P_1 \\ P_2 \\ Q \\ f_0 \end{bmatrix} \tag{S4}$$

where the amplitudes of the oscillator or radiation in the frequency domain are denoted by P_1, P_2, Q, and f_0. By substituting equation (S4) into equations (S1), (S2) and (S3), we arrive at the following:

$$\begin{bmatrix} \omega_1^2 - \omega^2 - i\gamma_1\omega \\ \omega_2^2 - \omega^2 - i\gamma_2\omega \\ \omega_m^2 - \omega^2 - i\gamma_m\omega \end{bmatrix} \bullet \begin{bmatrix} P_1 \\ P_2 \\ Q \end{bmatrix}^T = \begin{bmatrix} f_0 - c\mu P_2 \\ f_0 - c\mu P_1 - c_2\kappa_2 Q \\ -c_2\kappa_2 P_2 \end{bmatrix} \tag{S5}$$

Solving equation (S5) yields the resonant amplitudes of the modes in the coupled system as follows:

$$\begin{bmatrix} P_1 \\ P_2 \\ Q \end{bmatrix} = f_0 \begin{bmatrix} \dfrac{\Gamma_2 - \Gamma_3 - c\mu}{\Gamma_1(\Gamma_2 - \Gamma_3) - c^2\mu^2} \\ \dfrac{\Gamma_1 - c\mu}{\Gamma_1(\Gamma_2 - \Gamma_3) - c^2\mu^2} \\ -\dfrac{\Gamma_3}{c_2\kappa_2} \bullet \dfrac{\Gamma_1 - c\mu}{\Gamma_1(\Gamma_2 - \Gamma_3) - c^2\mu^2} \end{bmatrix} \tag{S6}$$

where

$$\begin{bmatrix} \Gamma_1 \\ \Gamma_2 \\ \Gamma_3 \end{bmatrix} = \begin{bmatrix} \omega_1^2 - \omega^2 - i\gamma_1\omega \\ \omega_2^2 - \omega^2 - i\gamma_2\omega \\ \dfrac{c_2\kappa_2^2}{\omega_m^2 - \omega^2 - i\gamma_m\omega} \end{bmatrix}$$

Since the dark oscillator lacks a dipole moment that matches the external field, it does not contribute to the surface current. Therefore, we can calculate the electric current density J as follows:

$$J = -i\omega n_s (P_1 + P_2) \tag{S7}$$

$$n_s (P_1 + P_2) = \varepsilon_0 \chi_{se}(\omega) E_s(\omega) \tag{S8}$$

According to equations (S18) and (S19), the surface conductivity can be determined as,

$$\sigma_{\text{se\_phonon}} = -in_s\omega \frac{\Gamma_1 + \Gamma_2 - \Gamma_3 - 2c\mu}{\Gamma_1(\Gamma_2 - \Gamma_3) - c^2\mu^2} \tag{S9}$$

We can calculate the scattering parameters, including reflection, transmission, and absorption, using equations (S9), (S10), and (S11). To illustrate, the reflection can be expressed as follows

$$R_{\text{phonon}} = |r|^2 = \left| -\frac{\xi\sigma_{\text{se\_phonon}}}{2+\xi\sigma_{\text{se\_phonon}}} \right|^2 \tag{S10}$$

To explore phonon-plasmon coupling, it is essential to calculate the signal strength of the phonon in the coupled system. This can be achieved by subtracting the background spectrum of the plasmon from the spectrum that contains the phonon signal, as follows:

$$\Delta R = R_0 - R_{\text{phonon}} = \left| -\frac{\xi\sigma_{\text{se}}}{2+\xi\sigma_{\text{se}}} \right|^2 - \left| -\frac{\xi\sigma_{\text{se\_phonon}}}{2+\xi\sigma_{\text{se\_phonon}}} \right|^2 \tag{S11}$$

where ω-ω_m is defined as the detuning δ of the plasmon-phonon coupling system.

Sensitivity refers to the deviation from the ideal slope of a characteristic curve, which measures how much an output changes in response to a change in the input quantity it measures. In the context of a phonon-plasmon coupling system, the output is the intensity of the reflection signal of the plasmonic resonance, while the input is the target molecules that cause a detuning between the plasmon and the phonon and thereby change the reflection intensity. Therefore, in our case, sensitivity can be calculated as follows:

$$S_{\text{phonon}} = \frac{d\Delta R}{d\delta} \tag{S12}$$

According to equation (S12), we can obtain Figure 2i in the main text.

B. Modeling the phonon polariton branches

The phonon polariton branches can be studied by analyzing two oscillators with eigenfrequencies ω+ and ω-, which are affected by the coupling strength between them. To observe how the vibration parameters are influenced by the coupling strength, we analyze the system of the two oscillators without any driving force. The resulting matrix equations describe the motion of the oscillators and can be expressed as follows:

$$\begin{bmatrix} \omega_2 - \omega - \dfrac{i\gamma_2\omega}{\omega_2+\omega} & \dfrac{\kappa_2}{\omega_2+\omega} \\ \dfrac{\kappa_2}{\omega_2+\omega} & \omega_{\mathrm{m}} - \omega - \dfrac{i\gamma_{\mathrm{m}}\omega}{\omega_{\mathrm{m}}+\omega} \end{bmatrix} \begin{bmatrix} p(t) \\ q(t) \end{bmatrix} = \mathbf{H} \bullet \begin{bmatrix} p(t) \\ q(t) \end{bmatrix} = \begin{bmatrix} 0 \\ 0 \end{bmatrix} \tag{S13}$$

where we can assume $|\omega-\omega_{2,\mathrm{m}}| \ll \omega$ when investigating the response of the system at or near resonance. To obtain a solution, we can solve the characteristic equation $|\mathbf{H}-\omega \boldsymbol{I}| = 0$, where $\boldsymbol{I}$ represents the identity matrix. The dressed frequencies of the system can be expressed as follows:

$$\omega_{\pm} = \frac{\omega_2+\omega_{\mathrm{m}}}{2} - i\frac{\gamma_2+\gamma_{\mathrm{m}}}{4} \pm \frac{1}{2}\sqrt{4g^2 + \left[(\omega_2-\omega_{\mathrm{m}}) - i\frac{\gamma_2-\gamma_{\mathrm{m}}}{2}\right]^2} \tag{S14}$$

where $g = \omega_+ - \omega_-$ is the frequency splitting. For a lossless ($\gamma_2=\gamma_{\mathrm{m}}=0$) system with $\omega_{\mathrm{m}} = \omega_0$, $\omega_2 = k$ and $g=\kappa\,\omega_0$, we can plot the bare and dressed frequencies of the coupling system, as shown in Supplementary Figure 6. As the coupling strength increases, the split between the upper and lower branches becomes more pronounced. In the strong coupling regime, a clear Rabi split can be observed. According to equation (S14), we can obtain Figure 1c in the main text.

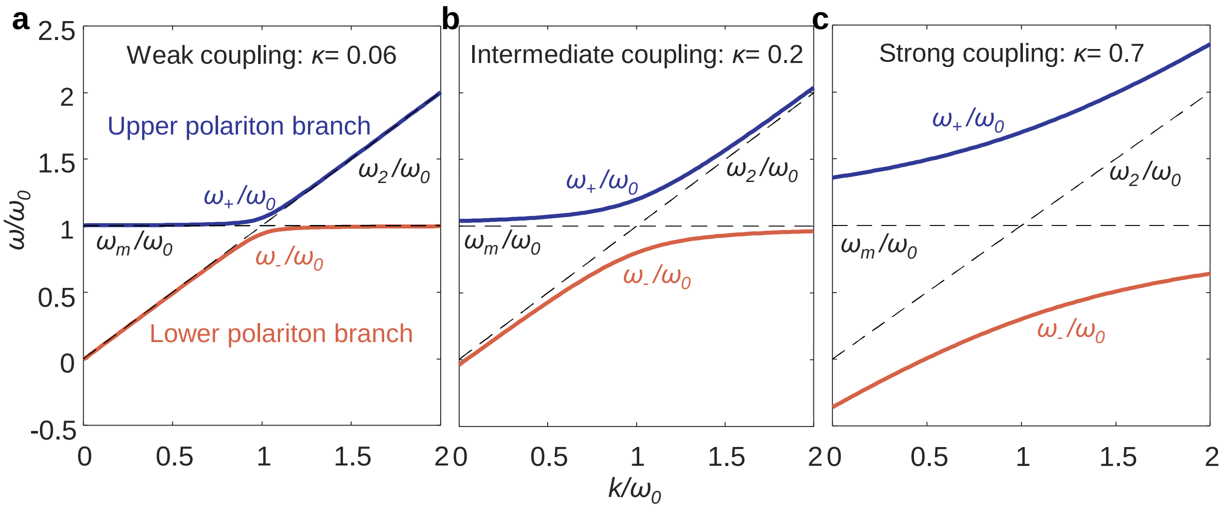

Supplementary Figure 6. Bare and dressed frequencies for the coupling system with different coupling strengths. a, Weak coupling with strength κ = 0.06. **b,** Intermediate coupling with κ = 0.2. **c,** Strong coupling with κ = 0.7.

Supplementary Note 3: More discussion on polariton-molecule interaction

Polaritons are hybrid quasiparticles formed due to the strong coupling of light to matter, including photon polaritons, exciton polaritons, and phonon polaritons [3]. In the IR region, the phonon polaritons and photon polaritons have received a great deal of attention, especially for sensing applications based on the interaction of polaritons and matter [4]. The large dipole moments and long lifetime of polaritons make them strongly interact with analytes in the surrounding environment. Therefore, polariton-based sensing sensors have the advantages of high sensitivity, low detection limit, nondestructiveness, and real-time detection.

The essence of the interaction between polaron and molecule is the interaction between polaron and refractive index and infrared vibration mode. In the case of the interaction with the refractive index, polaritons are sensitive to changes in the refractive index of the surrounding environment, which can be caused by the presence of analytes. When the refractive index of the environment changes, it affects the propagation of the electromagnetic field, and therefore the polariton properties, such as frequency shift and angle change. By measuring the changes in the polariton properties, we can detect the presence and concentration of analytes. In terms of the interaction with the vibrational modes of the molecules, the strong electric field component of the polariton. This interaction leads to changes in the vibrational properties of the molecules, such as changes in their intensity of vibration absorption. In the IR region, we know it as the Surface-Enhanced Infrared Absorption (SEIRA) effect.

Previous demonstrations have aimed to improve performance and push the technological frontier forward. In the case of polaronic refractive index-based sensors, the figure of merit (FOM) can be enhanced by reducing the bandwidth and increasing sensitivity. Methods to reduce bandwidth include utilizing bound states in the continuum (BIC) [5] and higher-order modes, among others [6]. Lowering the refractive index of the substrate is a way to increase sensitivity [7]. For SEIRA-based sensors, performance metrics such as LOD, sensitivity, and bandwidth can be improved by: 1) optimizing the design of the nanoantenna structure. For instance, reducing the distance between metamaterial pattern units can increase the near-field intensity, which is attributed to attractive electromagnetic force interactions [8]; 2) constructing a perfect absorber to increase near-field confinement and enhancement. In a dipole absorber, the electric and magnetic fields generate electric and magnetic dipoles within and between metal pattern layers, respectively

[9]; 3) improving the spatial overlap between analytes and enhanced near-field by integrating microfluidic techniques with nanoantennas [10]; 4) selecting appropriate plasmonic and dielectric materials. For instance, using Al as the plasmonic material can form a self-passivating oxide layer on the surface [11]; and 5) employing machine learning algorithms to simplify complex design and increase the types of detectable targets [12].

In our work, we develop a distinctive vibration, termed RI-dependent vibration, through the excitation of coupled surface plasmon-phonon polaritons. This unique vibration is a specific type of SPhP oscillation occurring within the reststrahlen band of polar dielectric crystals, exhibiting a notable sensitivity to the molecular refractive index. Notably, sensitivity to refractive index does not imply the simultaneous measurement of the real and imaginary parts of the composite refractive index in the same wavelength range. Our proposed method eliminates the need for any conversion using Kramers-Kronig relations and requires only 1 pixel to encompass refractive index information in our work. The physical mechanism behind the refractive index-dependent vibration response is the detuning of plasmonic resonance and SPhP mode in the reststrahlen band, as shown in Supplementary Figure 7. In our proposed stacked nanoantenna, the SPhP mode (Supplementary Figure 7a) and the plasmonic resonance mode (LSPP, Supplementary Figure 7b) are coupled to form an LSPP-SPhP mode hybridization. The spectra of the LSPP-SPhP nanoantenna are shown in Supplementary Figure 7c. where we can see a dip in the SPhP reststrahlen band (gray region). When loading a molecule, the real part of the molecule refractive index Δn will cause a redshift of the plasmonic resonance (LSPP) frequency. However, the SPhP mode frequency is fixed and not affected by Δn. This will cause a change in the detuning of plasmonic resonance and SPhP mode, as shown in Supplementary Figure 7d. Then, the change in the detuning further causes the change in the spectrum of SPhP mode in the reststrahlen band ΔI (because the coupling intensity of plasmonic resonance and SPhP mode is influenced). Supplementary Figure 7e shows the obtained spectrum of SPhP mode, which is determined by the real part of the molecule refractive index Δn. So we call it refractive index-dependent SPhP vibrations in the manuscript. Apparently, the frequency redshift-induced detuning of plasmonic resonance and SPhP mode is the key to this mechanism. Hopefully, our explanation has clarified the confusion for the reviewer.

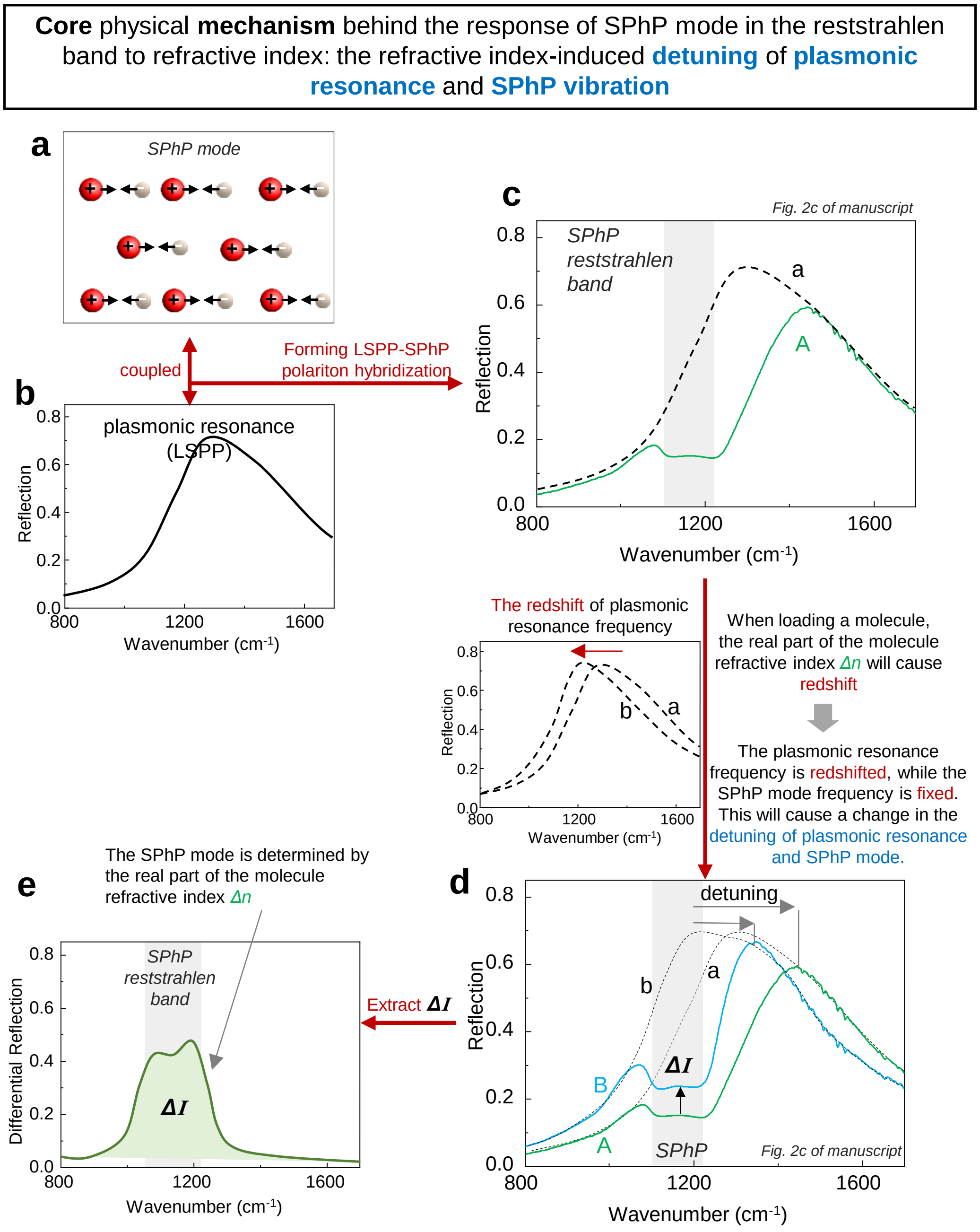

Supplementary Figure 7. The physical mechanism behind the response of SPhP mode in the reststrahlen band to refractive index. a, SPhP mode is coupled with (**b**) plasmonic resonance (LSPP). **c**, The spectrum of plasmonic resonance coupled with the SPhP mode. **d**, The spectra when loading a molecule with the real part of the molecule refractive index *Δn*. **e**, The extracted spectrum showing the SPhP mode.

Supplementary Note 4: Asymmetric least square algorithm for multispectral baseline fitting

The Asymmetric Least Squares algorithm is a potent tool for baseline correction. It is often used on a single spectrum, where the true baseline cannot be determined. In the case of multiple spectra, shared characteristics among them can be inferred to learn a gradually changing baseline, resulting in an accurate baseline. The particular technique involves estimating the baseline by penalizing the difference of the baseline correction signal, based on the resemblance between multiple spectra [13]. The optimization equation is based on the Whittaker Smoother and can be expressed as

$$\mathbf{z}_k = \arg\min_{z_k} \left\{ \sum_{u=1}^{m} \left\| (\mathbf{y}_u - \mathbf{z}_u) - \frac{1}{m} \sum_{v=1}^{m} (\mathbf{y}_v - \mathbf{z}_v) \right\|^2 \right.$$

$$\left. + \lambda \sum_{i=1}^{m} (\mathbf{y}_i - \mathbf{z}_i)^T Q_i (\mathbf{y}_i - \mathbf{z}_i) + \sum_{i=1}^{m} \mu_i \| D\mathbf{z}_i \|^2 \right\} \tag{S15}$$

where y_1, y_2, ..., y_m are spectral data with a length of m, and z_1, z_2, ..., z_m are target baselines. The first term on the right represents the difference between the corrected spectra pair-wise, and optimizing the baseline entails ensuring that each corrected spectrum is in close proximity to the average corrected spectrum. The second term is an asymmetric fitting error that measures the data. The final term imposes a smoothness constraint on the baseline. By introducing a relaxation factor a_u to each corrected spectrum, the corrected spectrum can deviate from the average corrected spectrum. Then, equation (S15) is extended to

$$(\mathbf{z}_k, a_k) = \arg\min_{z_k} \left\{ \sum_{u=1}^{m} \left\| (\mathbf{y}_u - \mathbf{z}_u) - a_u \frac{1}{m} \sum_{v=1}^{m} (\mathbf{y}_v - \mathbf{z}_v) \right\|^2 \right.$$

$$\left. + \lambda \sum_{i=1}^{m} (\mathbf{y}_i - \mathbf{z}_i)^T Q_i (\mathbf{y}_i - \mathbf{z}_i) + \sum_{i=1}^{m} \mu_i \| D\mathbf{z}_i \|^2 \right\} \tag{S16}$$

The solution to this equation is

$$\begin{cases} \mathbf{z}_k = \left[(m-\gamma_k)\mathbf{E} + \lambda' Q_k + \mu_k' D^T D \right]^{-1} \left[(m-\gamma_k) y_k - \gamma_k \sum_{i=1, i\neq k}^{m} (\mathbf{y}_i - \mathbf{z}_i) + \lambda' Q_k y_k \right] \\ a_k = \left(\theta^T \theta\right)^{-1} \theta^T (\mathbf{y}_k - \mathbf{z}_k) \end{cases} \quad \text{(S17)}$$

where **E** is the n×n identity matrix, and λ'=m λ, μ_k'=m μ_k.

After the initial value of each parameter is determined, the baseline can be fitted for multiple spectra according to equation (S17), and then the weight matrix is estimated as

$$Q_k^1(i,j) = \begin{cases} p, & y_k(i) > z_k^1(i), i = j \\ 1-p, & y_k(i) > z_k^1(i), i = j \\ 0, & \text{otherwise} \end{cases} \quad \text{(S18)}$$

Then, we calculate the baseline using equation (S17) in Matlab software. Supplementary Figure 8a,b illustrates the optimization process of baseline fitting by adjusting the weight parameter *P* and regularization parameter μ. It is observed that *P* can control the position of the baseline while μ represents the similarity constraint. By iteratively updating the baseline and relaxation factor based on the equation (S17), the final baseline can be derived. Supplementary Figure 8c demonstrates the application of this method for multispectral baseline fitting and extraction of IR fingerprints of bovine serum albumin (BSA).

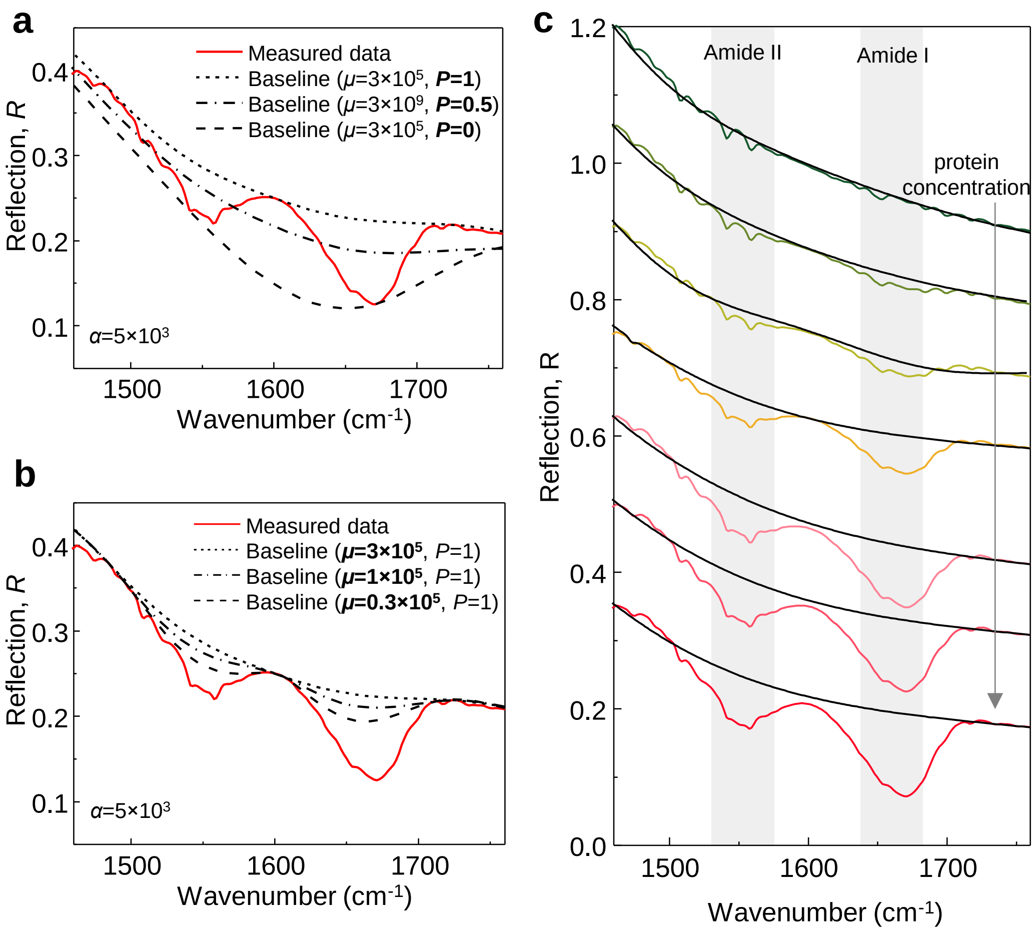

Supplementary Figure 8. Asymmetric least square algorithm for multispectral baseline fitting and IR fingerprints extraction. a, The effect of the weight parameter p and **b,** the regularization parameter β on the fitting result. **c,** Baseline correction for extracting IR fingerprint signals of amide I and II of protein BSA using the asymmetric least square algorithm. Regularization parameter λ=5000.

Supplementary Note 5: DNN model training and validation

A. DNN model for SP-PhP platform

Data preprocessing is a crucial step in DNN model training as it helps to ensure that the model is able to learn from spectral data, converge faster, and achieve high accuracy. We conduct two types of experiments to generate training data: one involves changing the amount of only one analyte per measurement (univariate experiment), while the other involves changing all analytes simultaneously (multivariate experiment).

In univariate experiments, the objective is to determine the contribution of each analyte to the refractive index change, that is, to establish the relationship between the vibration of the analyte and the RI-determined SPhP vibration. This information allows the model to decouple overlapping vibrations in the mixture based on their respective contributions. Figure 5a in the manuscript shows the differential absorbance spectra versus wavenumber and concentration when using the SP-PhP platform to detect one analyte at a time. Then, regression methods are used to extract the vibrations of the analytes and the SPhP signals. The resulting regression signal represents the intensity of vibration for both the analytes and SPhP. As observed, the intensity relationship between the analyte and the SPhP is unique. For instance, when the regression signal of SPhP reaches 1, the regression signals of D-gluconic-1,5-lactone and glucose are 0.8 and 0.7, respectively. This ratio, combined with the total SPhP signal intensity in their mixture, allows the calculation of their respective quantities in the mixture, thus decoupling from overlapping vibrations. In practical applications, their mathematical relationship is more complicated. but by training the DNN mode to learn the relationship behind the data, we can realize complex applications such as the glucose enzymatic reaction. The methodology used to compute regression signals from spectra has been previously reported in the literature [14]. In the multivariate experiment, all analytes are detected at the same time, and the total contribution of all analytes is obtained accordingly (Supplementary Figure 9a,b).

The training and validation dataset comprises 20,156,640 spectrotemporal data points obtained from both univariate and multivariate experiments. We repeated the measurement for each participant in the reaction. We also repeated the measurement for all participants of the reaction after mixing. The training and validation sets are split by a ratio of 4:1, that is, 80% is for

the training data set, and 20% is for the testing data set. In this work, the DNN models for regression are developed using Keras, a high-level neural networks API written in Python that allows for easy and flexible model construction. The architecture of the DNN model consists of fully connected layers, also called dense layers. The activation function used in the layers is ReLu, which stands for Rectified Linear Unit, a popular choice for deep learning models because it is computationally efficient and allows for faster convergence during training. The DNN model is composed of input, hidden, and output layers. The input layer has 1714 nodes, corresponding to the wavenumber points of the spectrum, while the output layer has 5 nodes, matching the vibration numbers of the GER reactants. The model also includes two hidden layers, each with 20 nodes, designed to extract high-level features from the input data. These layers enable the model to learn complex relationships and make accurate predictions.

The loss function used in the model is mean square error (MSE), which measures the average squared difference between the predicted and actual values. The optimizer used in the final model is Adam, which is a powerful optimization algorithm that adapts the learning rate based on the gradient of the loss function, allowing for more efficient training of the model. During training, the model adjusts the weights and biases in the layers to minimize the loss function and improve the accuracy of the predictions. Once the training is complete, the model can be evaluated on a test dataset to assess its performance on new data. The result shows that the accuracy of the trained model reaches 92% (Supplementary Figure 9c,d).

For a machine learning model, its generality refers to the ability to generalize patterns and knowledge from the training data to make accurate predictions or decisions on unseen or new data. In our case, we input a new measurement data set to predict the bioreaction process. Besides, during the training process, the data is split by a ratio of 4:1 (20% is for the testing data set). The results show that the identification accuracy is 92%. Therefore, from the perspective of the machine learning field, our model exhibits good generality. When the target molecules are changed to other molecules, the refractive index of these new molecules changes but remains detectable by the antenna. For the part of machine learning algorithms, when the target molecules are changed to other molecules, the structure of the algorithm is general and still applies. We only need to retrain the machine learning model using the new data from our nanophotonic device. Overall, our methodology and model are general and not limited to specific molecules.

B. DNN model for nanorod platform

The DNN model for the nanorod platform is developed using a similar method. Notably, there are no RI-determined SPhP vibrations to quantify the individual contributions of each analyte to refractive index change, as shown in Supplementary Figure 10. Hence, it becomes challenging to decouple analyte vibrational modes when they significantly overlap with each other. This is the reason for the poor performance of the nanorod platform in the demonstration of glucose enzymatic reaction (Figure 5e of the main text). The DNN model incorrectly assigns the vibration signal of H_2O_2 to GOD because it cannot distinguish between the amide vibration of GOD and the H-O vibration of H_2O_2.

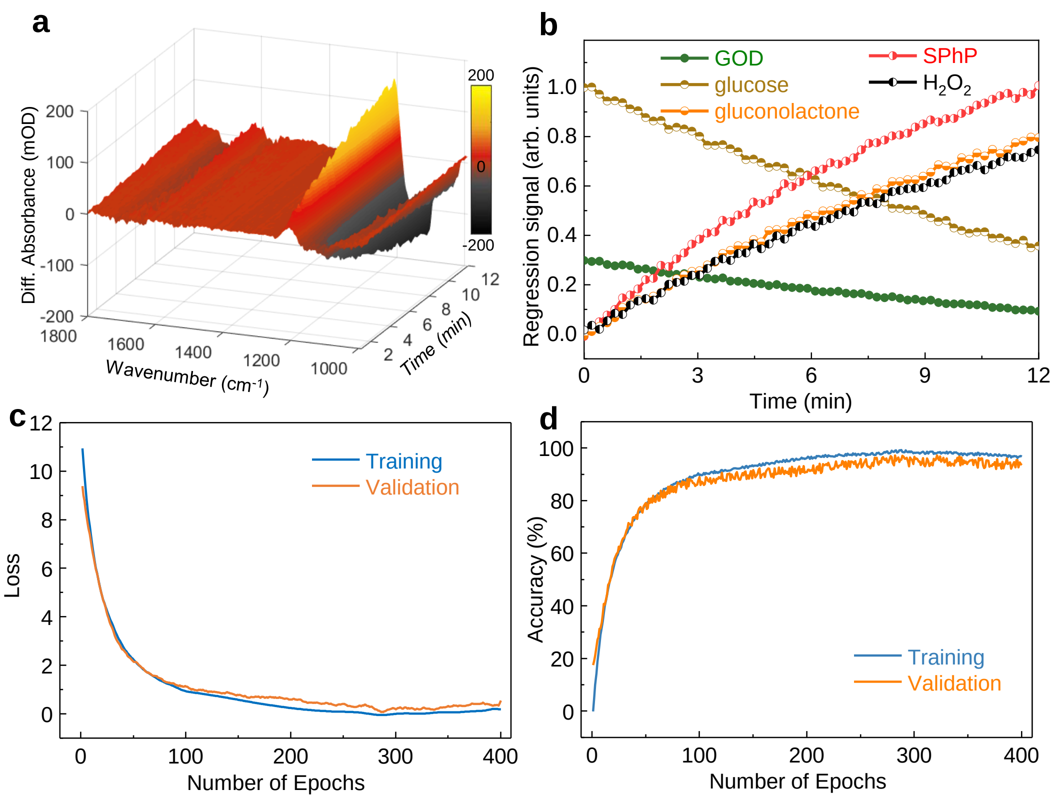

Supplementary Figure 9. Training results of the DNN model. a, Real-time 3D plots of differential absorbance spectra when all analytes are detected by the SP-PhP platform at the same time. **b,** Corresponding regression curve. **c,** Loss (mean square error) and **d,** accuracy of the DNN model in the training and validation process with the increase in the number of epochs.

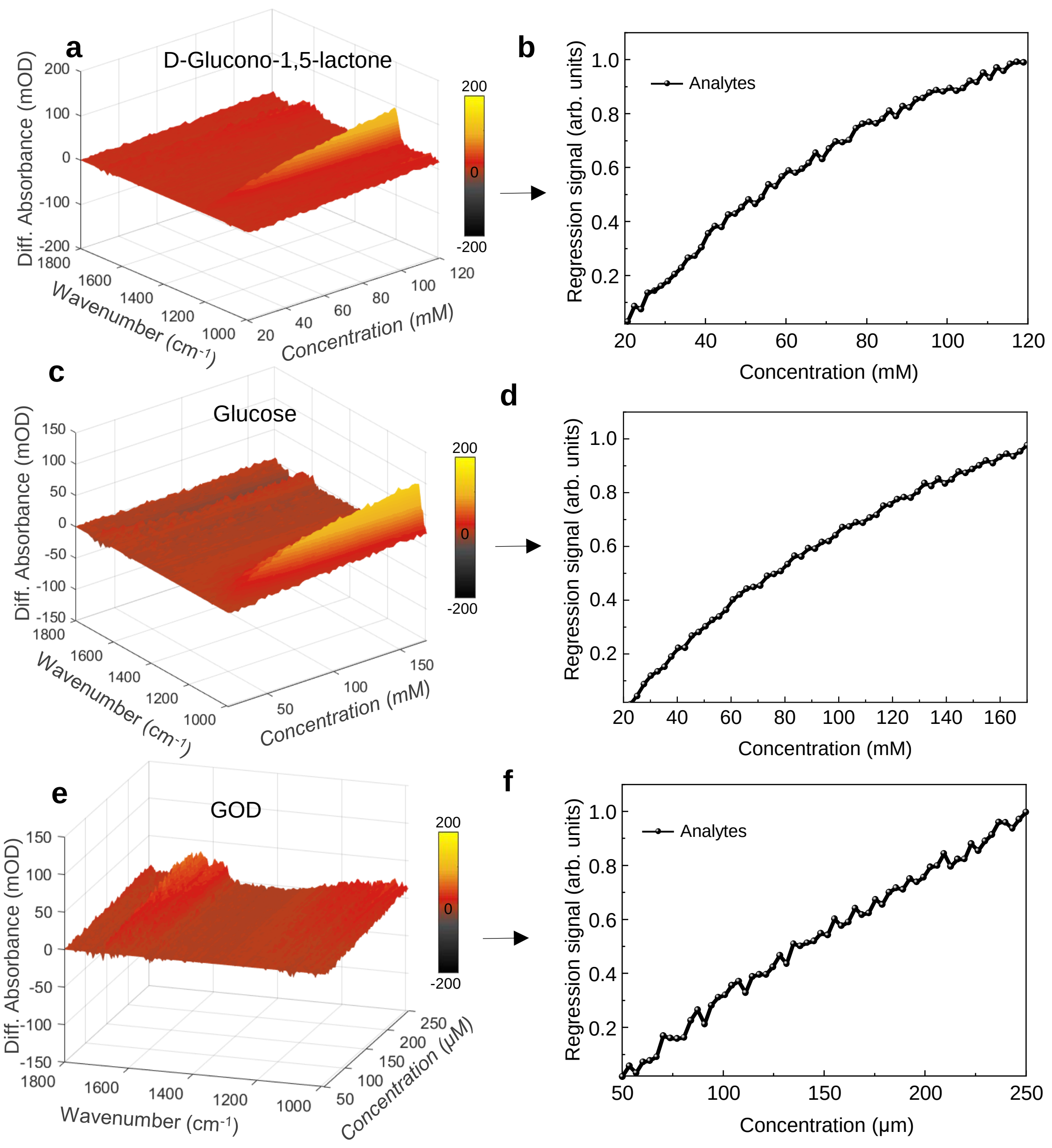

Supplementary Figure 10. Nanorod platform Dataset with one analyte feature variation for DNN training. a,c,e, Real-time 3D plots of differential absorbance spectra versus wavenumber and concentration when using the nanorod platform to detect one analyte at a time, including (**a**) glucono-1,5-lactone, (**c**) glucose, (**e**) GOD. **b,d,f,** Corresponding regression curve. As observed, the difference between the nanorod platform and the SP-PhP platform is that there is no SPhP signal for the DNN model to refer to. That is, the contribution of each analyte to refractive index change is unknown.

Supplementary Note 6: Kramers–Kronig relations

The real part and imaginary part of the complex refractive index are linked by Kramers-Kronig relations. Next, we use the Kramers-Kronig relations to calculate the real part of the complex refractive index. Supplementary Figure 11a is the refractive index *n* and extinction *k* (the imaginary part of the complex refractive index) of glucose (Corresponding to Figure 3a-III of the manuscript). Supplementary Figure 11b shows the simulated results of two bare devices with different resonance frequencies, denoted as Device 1 and Device 2. Then we put them into the FDTD simulations. Supplementary Figure 11c is the obtained reflection result of SEIRAS Device 1 when coupled with glucose molecules. Next, we extract the differential reflectance spectra through baseline calibration (Supplementary Figure 11d), which is the SEIRAS signal reflecting the imaginary part of the complex refractive index. Subsequently, we calculate the real part n using Kramers–Kronig relations, as shown below:

$$n(\omega) = n(\infty) + \frac{2}{\pi}\int_0^\infty \frac{k(x)-k(\omega)}{x^2-\omega^2}dx \qquad \text{(S19)}$$

where ω is the frequency. After calculation in Matlab software, the n is obtained and plotted in Supplementary Figure 11e. During the calculation, we rescale the differential reflectance spectra since the SEIRAS signal represents an amplified value of the imaginary part. Then, we calculate *n* using data from Device 2 (Supplementary Figure 11f-h). The resonance between SEIRAS Device 1 and Device 2 is different, so the detuning of molecule vibration and SEIRAS chip resonances is different. For comparison, we plot the original *n* and the calculated *n* from Device 1,2 together, as shown in Supplementary Figure 11i.

As observed, some features are absent in the calculated real part *n* by using Kramers–Kronig relations. In Region A of Supplementary Figure 11i, it is evident that certain peaks present in the original *n* curve are not observable in the calculated *n* curves. Besides, there exist substantial disparities in both shape and magnitude between the original curve and the calculated curve (gap B in Supplementary Figure 11i). Finally, there are also discrepancies between calculated curves obtained with different devices as evident from the orange and blue solid curves in Supplementary Figure 11i.

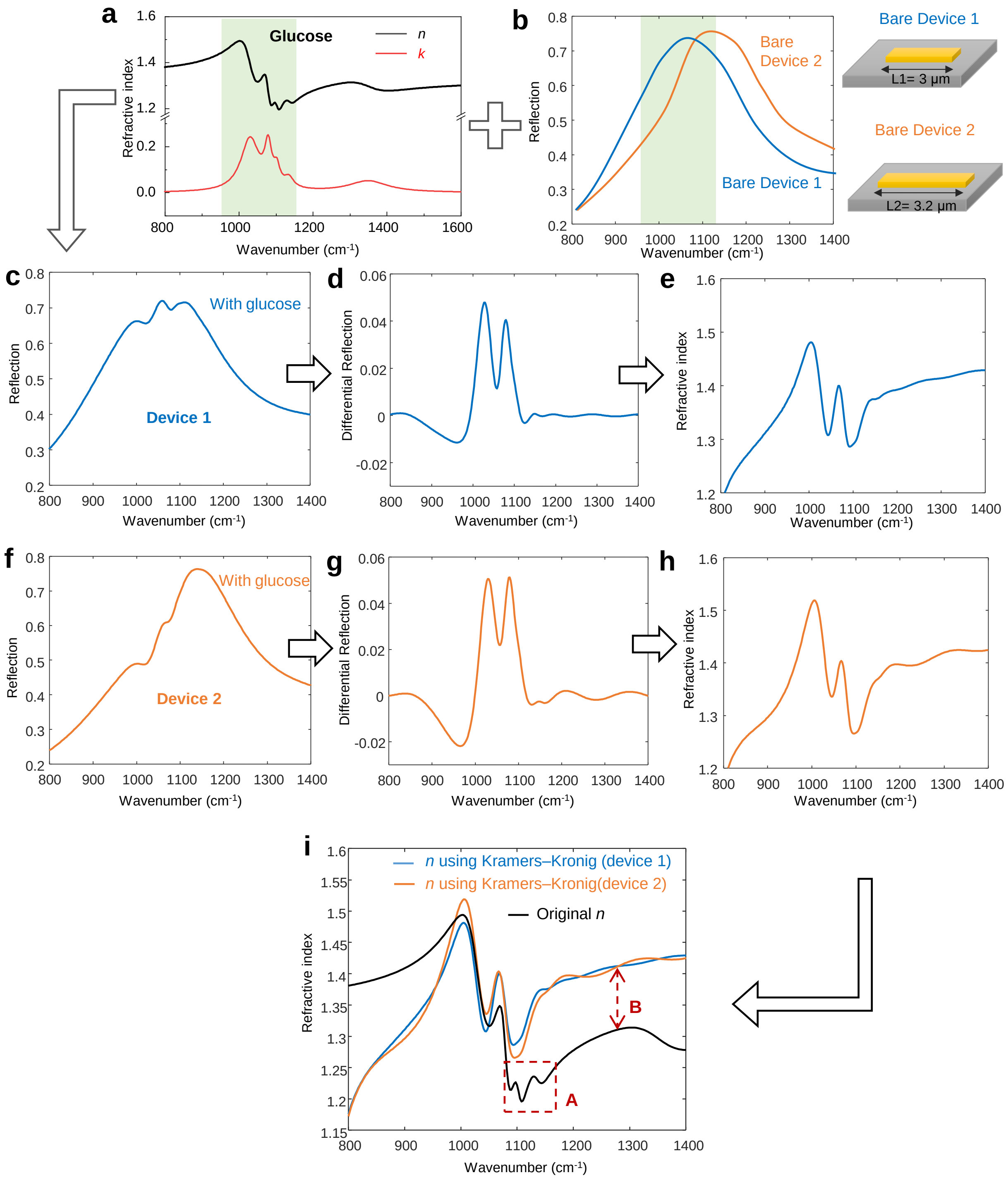

Supplementary Figure 11. Calculation of refractive index using Kramers-Kronig relations. **a,** Original refractive index *n* and extinction *k* of glucose. **b**, Bare plasmonic device for SEIRAS. The resonance frequency between Device 1 and Device 2 is different. **c**, Simulated reflection result of SEIRAS Device 1 coupled with glucose molecules. **d**, Extracted differential reflectance spectra. **e**, Calculated n using Kramers-Kronig relations. **f-h**, Calculation of n using the data from another SEIRAS Device 2. **i**, Comparison of original *n* and calculated *n* using Kramers-Kronig relations.

Supplementary Note 7: Complex refractive index calculation

The complex permittivity of an analyte determines its optical parameters, including reflectance and absorptivity. By measuring the infrared absorption of an analyte, we can calculate its complex permittivity through inverse calculation. The Drude-Lorentz model is a mathematical approach used to calculate the complex dielectric properties of analytes, which can be expressed as

$$\varepsilon(\omega) = \varepsilon_\infty + \sum_i^n \frac{S_i}{\omega_{0i}^2 - \omega^2 - j\omega\gamma_i} \tag{x}$$

Here, ε_∞ represents the high-frequency constant term, S_i denotes the oscillator strength, ω_0 is the oscillator resonance frequency, and γ represents the damping frequency. The Fresnel formula expresses the relationship between reflectivity and dielectric function for normal incidence configuration as follows

$$R = \left| \frac{1-\sqrt{\varepsilon}}{1+\sqrt{\varepsilon}} \right| \tag{x}$$

By applying the equations presented above, we can establish a relationship between the spectrum and the complex permittivity of an analyte. We can then use the absorption spectrum of the analyte to calculate its complex permittivity (see Supplementary Figure 12a,b). The fitting parameters are listed in Supplementary Table 1. Then, we can calculate the complex refractive index using the equation $n^2 = \varepsilon$, as shown in Supplementary Figure 12c. To confirm the accuracy of our fitting, we imported the calculated complex refractive index into FDTD software and obtained simulated results, which are shown in Supplementary Figure 12d. As demonstrated, our simulation results closely match the measured absorption spectra, validating the accuracy of our approach.

Supplementary Table 1. Drude-Lorentz model fitting parameters

Parameter \ *i*	**1**	**2**	**3**	**4**	**5**
ω_i (cm^{-1})	1028	1078	1102	1135	1350
S_i (cm^{-1})	14.14	10.24	7.74	7.74	12.24
γ_i (cm^{-1})	60	23	19	30	130

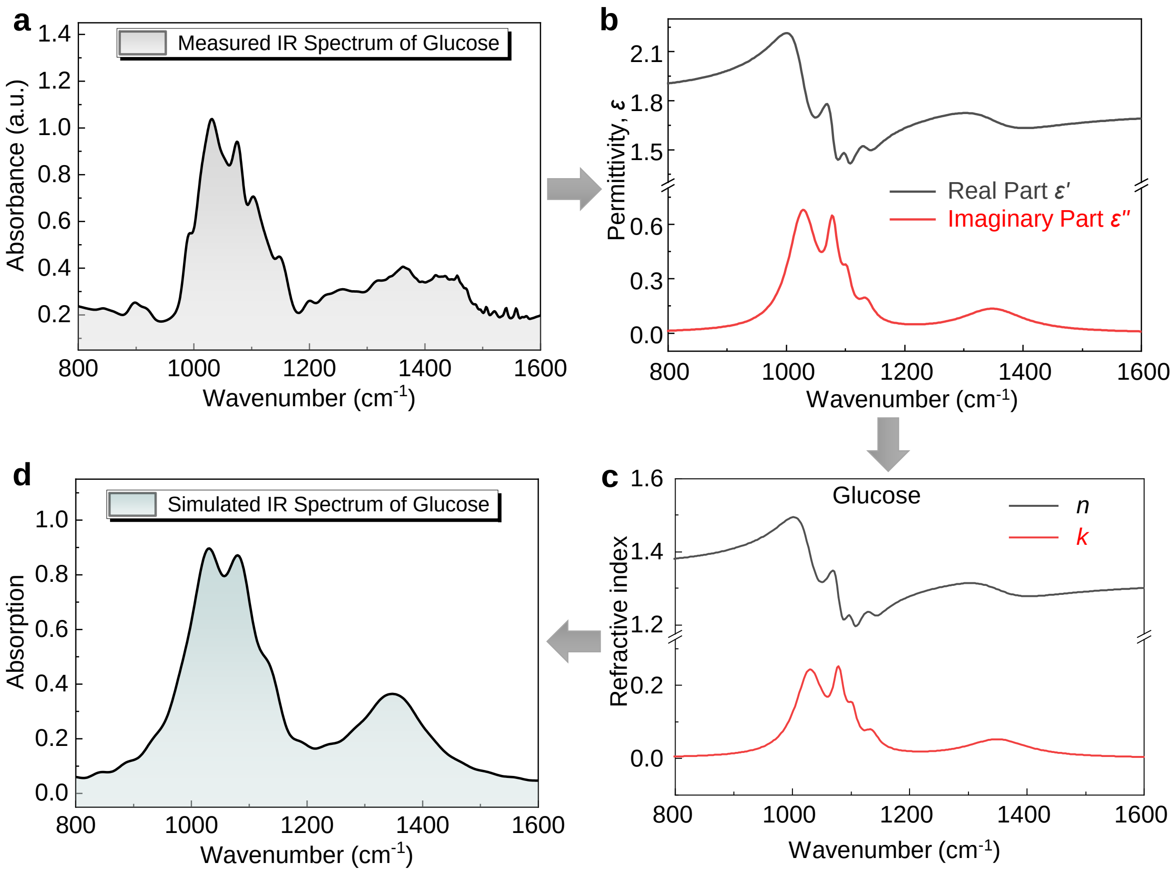

Supplementary Figure 12. Calculation of complex refractive index**. a,** Measured absorption spectra of glucose film. **b,** Real (black curve) and imaginary (red curve) parts of glucose calculated using the Drude permittivity model. **c,** Complex refractive index of glucose extracted from (**b**). **d,** FDTD simulation results using the calculated permittivity to verify the correctness of the calculated permittivity.

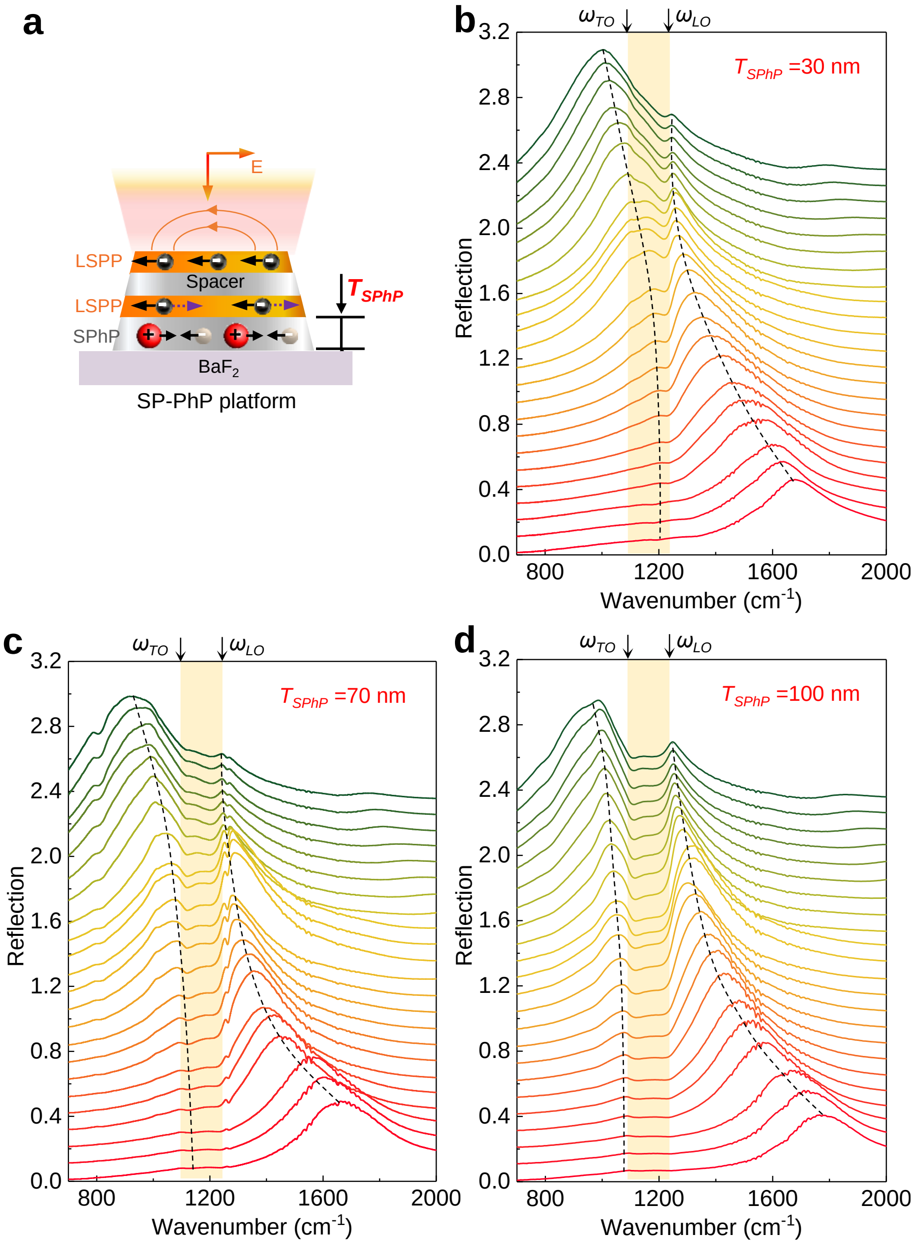

Supplementary Figure 13. Thickness-dependent transition of plasmon-phonon coupling. a, Schematic of the SP-PhP platform for investigating plasmon–phonon coupling. **b,** Experimental reflection spectra of the platform with SPhP layer thickness T_{SPhP} = 30 nm and antenna length ranging from 2.2 to 4.2 μm, which corresponds to the plasmonic resonances from 900 to 1700 cm^{-1}. The resonance range covers the reststrahlen band of the SPhP mode from ω_{TO} to ω_{LO}. **c,** The response of the platform when T_{SPhP} = 70 nm and **d,** T_{SPhP} = 100 nm. As observed, the strength of plasmon-phonon coupling can be controlled by changing the thickness of the SPhP antenna.

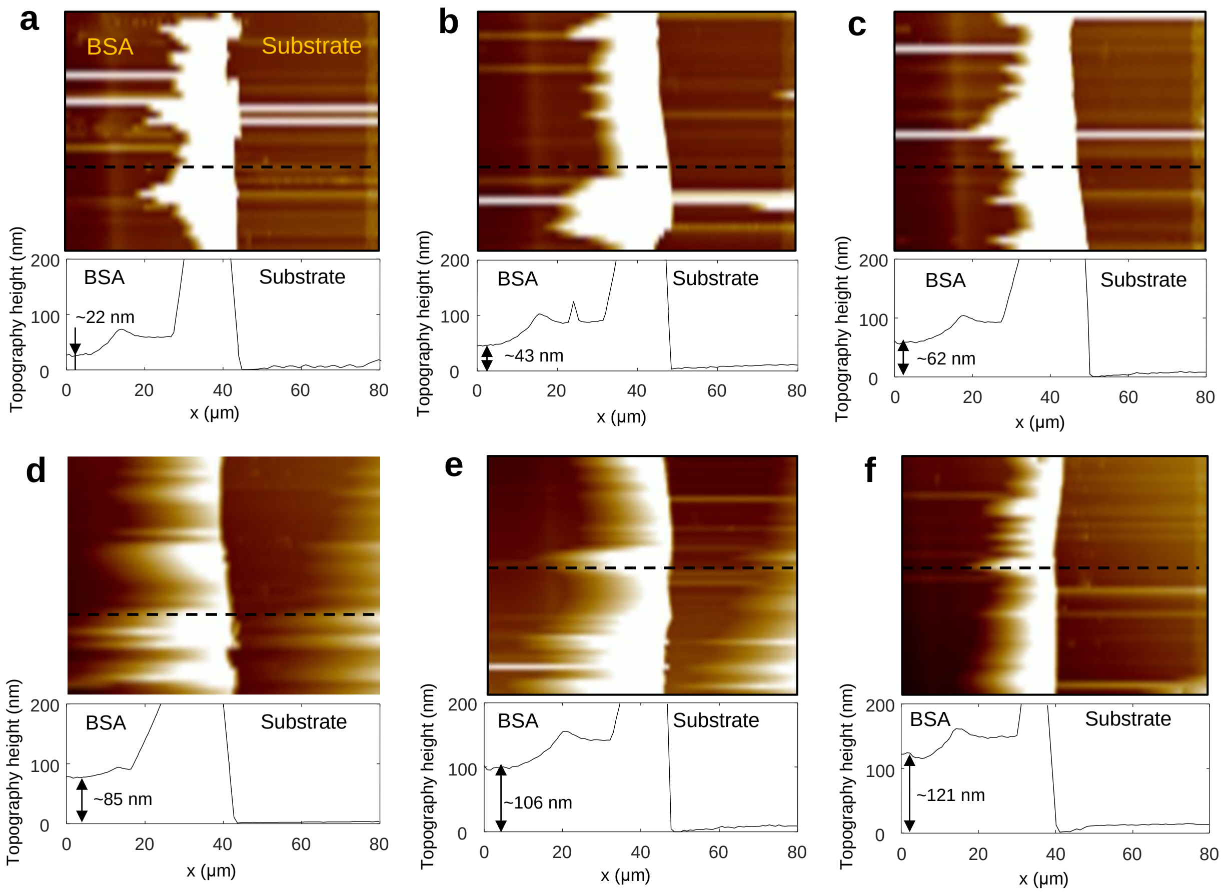

Supplementary Figure 14. BSA thickness characterization using atomic force microscopy (AFM). a, BSA thickness of 22 nm. **b**, BSA thickness of 43 nm. **c**, BSA thickness of 62 nm. **d**, BSA thickness of 85 nm. **e**, BSA thickness of 106 nm. **f**, BSA thickness of 121 nm. The thickness is controlled by the coating speed and BSA concentrations. During the spin-coating process, part of the substrate was protected by polyimide tape. After spin coating, the tape was removed and measured using AFM. The measured results correspond to Figure 3g of the main text. The transition region between the protein film and the substrate was shown to be abnormally thick. Because BSA accumulated at the edge of the tape during spin coating.

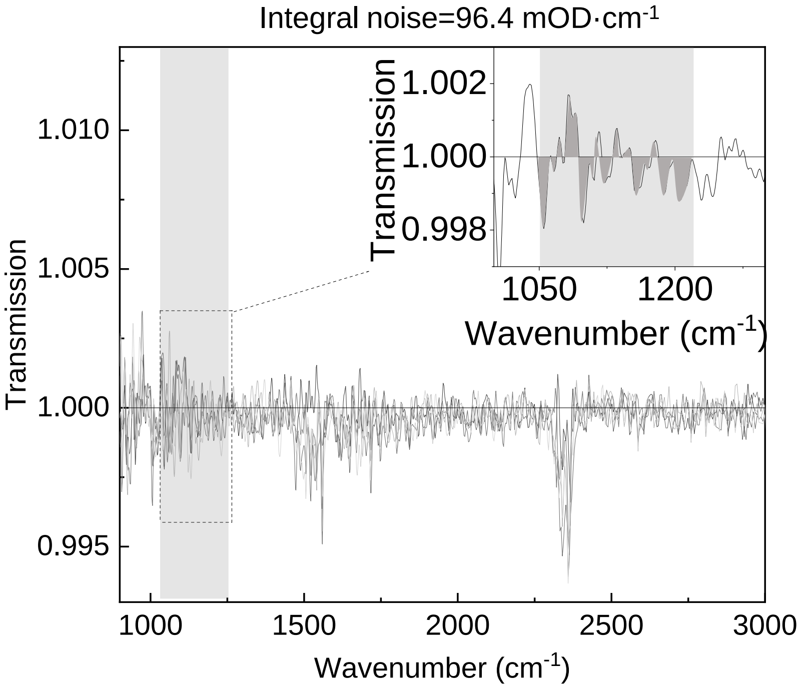

Supplementary Figure 15. Noise analysis of the experimental setup. The noise spectra were obtained on the blank BaF_2 substrate without nanoantennas. The Integral noise is calculated by $N = \int_{1050}^{1250} \left| T - T_{aver} \right| dx$, where T is the noise spectra. According to the calculation, the average integral noise of 20 groups is 96.4 mOD·cm^{-1}. An example of integrating a noise spectrum is shown in the inset.

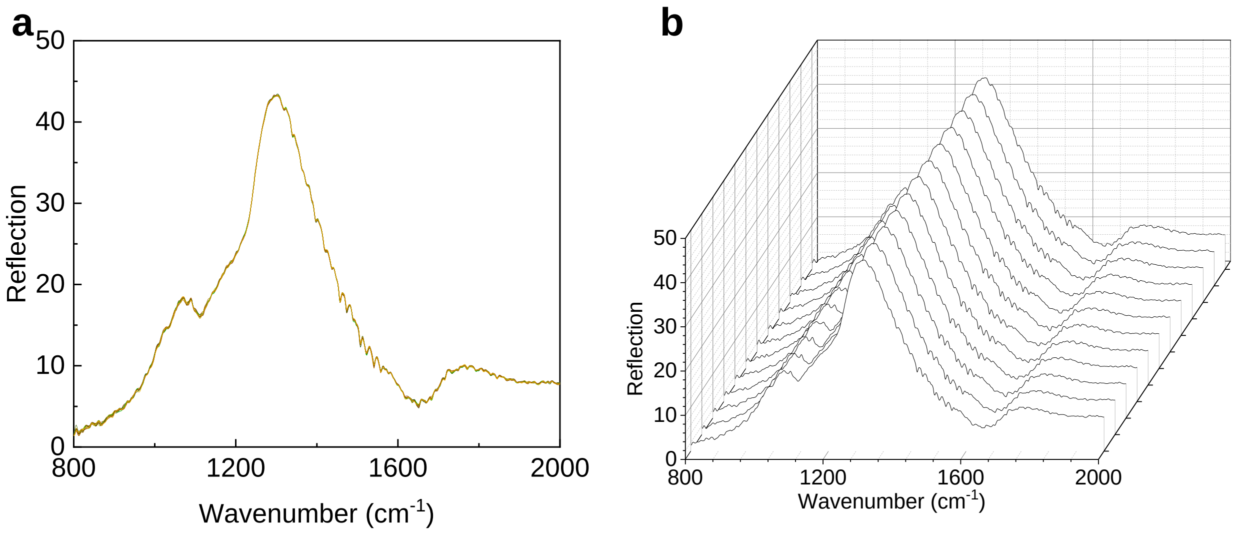

Supplementary Figure 16. Environmental disturbances. a, 2D views of reflection spectra of our device with varying environmental humidity (30%-60%RH) and temperature (20-30 Celsius). **b**, 3D views of the spectra in (a). As observed, the spectra are basically the same. The main reason is that we measure the background spectrum before each measurement. The background spectrum contains contributions from the surrounding environment, such as water vapor and carbon dioxide present in the air. These atmospheric components can be eliminated by measuring the background spectrum.

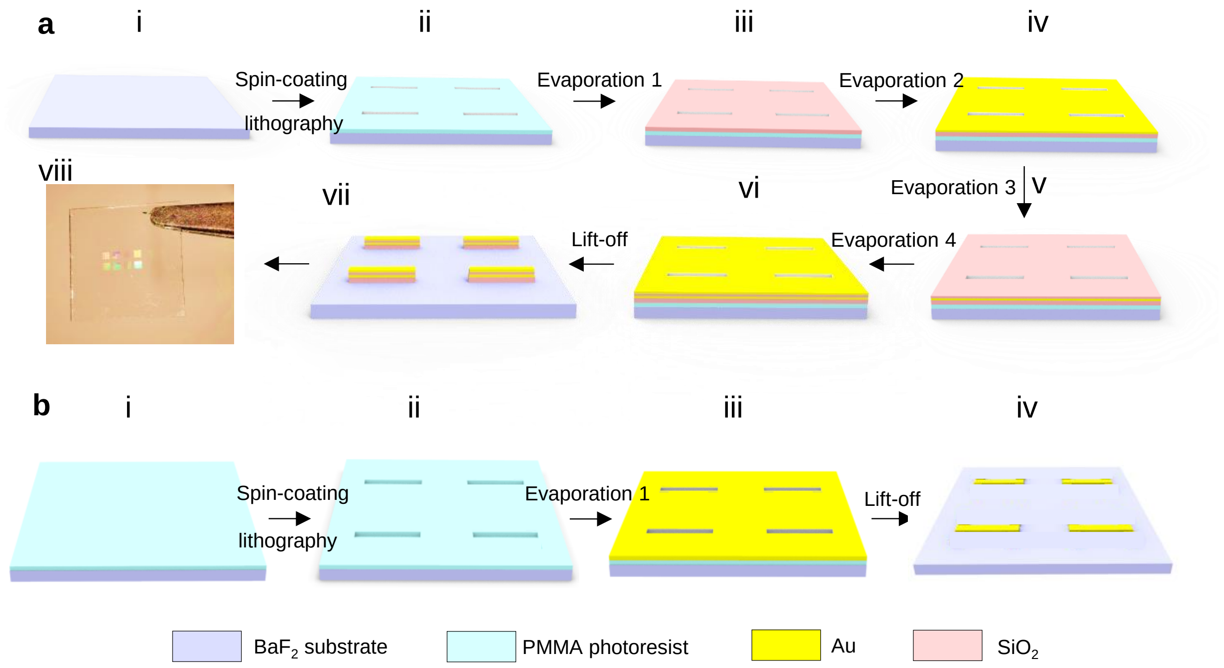

Supplementary Figure 17. Nanofabrication processes. a, The nanofabrication of the SP-PhP platform. i: The procedure started by cleaning a BaF_2 wafer in acetone using ultrasonic treatment for 10 minutes. The wafer was then rinsed in isopropanol and dried with nitrogen before undergoing 5 minutes of oxygen plasma treatment. ii: A 400 nm thick layer of PMMA e-beam lithography resist (950 PMMA A5) was spin-coated at 3000 rpm, followed by the spin-coating of a commercial electron-conducting polymer. Then it was exposed using e-beam lithography and developed using deionized water, MIBK/IPA (1:3) mixture, and isopropanol. iii-vi: SiO_2/Ti/Au/Ti/SiO_2/ Ti/Au layers of desired thickness were sequentially deposited using e-beam evaporation. vii: The unexposed resist was removed using a lift-off process. viii: The final SP-PhP chip. **b,** The nanofabrication of the nanorod platform. i: Wafer Cleaning. ii: Spin-coating PMMA e-beam lithography resists and electron-conducting polymer, followed by e-beam lithography and developing. iii: Deposit Ti/Au layers using e-beam evaporation. iv: The final nanorod chip was obtained using a lift-off process.

Supplementary Table 2. Mode splitting of SP-PhP platform

**Parameters**	**Value**
Thickness of the SPhP layer (nm)	100
TO phonon frequency, ω_{TO} (cm^{-1})	1050
Linewidth of the bare antenna without SPhP layer at TO phonon frequency (cm^{-1})	550
Average of bare antenna and TO phonon linewidths, γ_{avg} (cm^{-1})	313
Normal mode splitting, $2g$ (cm^{-1})	290 cm^{-1}
Normalized mode splitting, $2g/\gamma_{avg}$	0.92
Normalized coupling strength, $\eta=g/\omega_{TO}$	0.138

Linewidth is defined as the full width at half maximum.

Supplementary Table 3. Comparison of infrared antenna-based biosensors

Optical element	Detection mechanism	FWHM (μm)	Analyte	Optical properties	Signal type	Remarks
Metal nanorod[15]	Refractive index-sensitive LSPP mode	~0.1	PBS buffer	Refractive index	Frequency shift	30,000 nm/RIU sensitivity
Hyperbolic metamaterials[16]	Refractive index-sensitive LSPP mode	~0.03	BSA, biotin	Refractive index	Frequency shift	30,000 nm/RIU sensitivity
BIC-enabled metasurfaces[5]	Refractive index-sensitive LSPP mode	~0.02	Glycerol	Refractive index	Frequency shift	486 nm/RIU
Metal mushroom arrays[17]	Refractive index-sensitive LSPP mode	~0.01	Cytochrome, Alpha-fetoprotein	Refractive index	Frequency shift	1,010 nm/RIU
2D material[18]	Refractive index-sensitive SPP mode	/	miRNA	Refractive index	Angle shift	171°/RIU
Metal micropillars[19]	SEIRA	~0.75	miRNA-155	Absorption	Absorption change	1.162%/pM sensitivity
Dual-resonant nanorod[14]	SEIRA	~0.9 ~1.3	Lipid, melittin	Absorption	Absorption change	1000-fold near-field intensity enhancement; Dynamic monitoring;
Dielectric BIC metasurfaces[20]	SEIRA	Pixel: ~0.002	Single-layer graphene	Absorption	Absorption change	Extremely narrow bandwidth
Graphene acoustic plasmon resonator[21]	SEIRA	~1.7	Silk, silicon oxide	Absorption	Absorption change	Ångström-thick film sensing
Multiplexed nanoantenna[1]	SEIRA	3	Alcohol mixtures	Absorption	Absorption change	Ultra-broadband; 100% identification accuracy
Thin h-BN layer	Phonon-molecule coupling	\	CBP	Absorption	Absorption change	vibrational strong coupling
Quartz micropillars[22]	Phonon-molecule coupling	0.17	4-nitrobenzyl alcohol	Absorption	Absorption change	2.5 mM
Phononic wavelength[23]	Refractive index-sensitive Fano resonance	0.12	Refractive index	Refractive index	Frequency shift	377.2 nm/RIU
SP-PhP platform (**This work**)	Refractive index-induced detuning; SEIRA	3, Bare antenna: 602 cm^{-1}	Glucose enzymatic reaction	Refractive index **& Absorption**	**Amplitude change**	Joint baseline correction; Enable to decouple overlapping vibrations;

FWHM, full width at half-maximum. **SEIRA**, surface-enhanced infrared absorption.

BIC, bound states in the continuum. **LoD**, limit of detection. **CPB**, 4,4’-bis(N-carbazolyl)-1,1’-biphenyl.

h-BN, hexagonal boron nitride.

Supplementary References

1. Ren, Z., Zhang, Z., Wei, J., Dong, B.& Lee, C. Wavelength-multiplexed hook nanoantennas for machine learning enabled mid-infrared spectroscopy. *Nat. Commun.* **13**, 3859 (2022).

2. Tittl, A., Leitis, A., Liu, M., Yesilkoy, F., Choi, D.-Y., *et al.* Imaging-based molecular barcoding with pixelated dielectric metasurfaces. *Science* **360**, 1105-1109 (2018).

3. Caldwell, J. D., Lindsay, L., Giannini, V., Vurgaftman, I., Reinecke, T. L., *et al.* Low-loss, infrared and terahertz nanophotonics using surface phonon polaritons. *Nanophotonics* **4**, 44-68 (2015).

4. Yoo, D., de León-Pérez, F., Pelton, M., Lee, I.-H., Mohr, D. A., *et al.* Ultrastrong plasmon–phonon coupling via epsilon-near-zero nanocavities. *Nat. Photonics* **15**, 125-130 (2020).

5. Wang, Z., Sun, J., Li, J., Wang, L., Li, Z., *et al.* Customizing 2.5D Out-of-Plane Architectures for Robust Plasmonic Bound-States-in-the-Continuum Metasurfaces. *Adv. Sci.* **10**, e2206236 (2023).

6. Linnenbank, H., Grynko, Y., Forstner, J.& Linden, S. Second harmonic generation spectroscopy on hybrid plasmonic/dielectric nanoantennas. *Light Sci Appl* **5**, e16013 (2016).

7. Wu, X., Quan, B., Pan, X., Xu, X., Lu, X., *et al.* Alkanethiol-functionalized terahertz metamaterial as label-free, highly-sensitive and specific biosensor. *Biosens. Bioelectron.* **42**, 626-631 (2013).

8. Brown, L. V., Yang, X., Zhao, K., Zheng, B. Y., Nordlander, P., *et al.* Fan-Shaped Gold Nanoantennas above Reflective Substrates for Surface-Enhanced Infrared Absorption (SEIRA). *Nano Lett.* **15**, 1272-1280 (2015).

9. Hasan, D.& Lee, C. Hybrid Metamaterial Absorber Platform for Sensing of CO2 Gas at Mid-IR. *Adv. Sci.* **5**, 1700581 (2018).

10. Hu, X., Xu, G. Q., Wen, L., Wang, H. C., Zhao, Y. C., *et al.* Metamaterial absorber integrated microfluidic terahertz sensors. *Laser Photonics Rev.* **10**, 962-969 (2016).

11. Cerjan, B., Yang, X., Nordlander, P.& Halas, N. J. Asymmetric aluminum antennas for self-calibrating surface-enhanced infrared absorption spectroscopy. *ACS Photonics* **3**, 354-360 (2016).

12. Meng, J., Cadusch, J. J.& Crozier, K. B. Plasmonic Mid-Infrared Filter Array-Detector Array Chemical Classifier Based on Machine Learning. *ACS Photonics* **8**, 648-657 (2021).

13. Peng, J., Peng, S., Jiang, A., Wei, J., Li, C., *et al.* Asymmetric least squares for multiple spectra baseline correction. *Anal. Chim. Acta* **683**, 63-68 (2010).

14. Rodrigo, D., Tittl, A., Ait-Bouziad, N., John-Herpin, A., Limaj, O., *et al.* Resolving molecule-specific information in dynamic lipid membrane processes with multi-resonant infrared metasurfaces. *Nat. Commun.* **9**, 2160 (2018).

15. Kabashin, A. V., Evans, P., Pastkovsky, S., Hendren, W., Wurtz, G. A., *et al.* Plasmonic nanorod metamaterials for biosensing. *Nat. Mater.* **8**, 867-871 (2009).

16. Sreekanth, K. V., Alapan, Y., ElKabbash, M., Ilker, E., Hinczewski, M., *et al.* Extreme sensitivity biosensing platform based on hyperbolic metamaterials. *Nat. Mater.* **15**, 621-627 (2016).

17. Shen, Y., Zhou, J., Liu, T., Tao, Y., Jiang, R., *et al.* Plasmonic gold mushroom arrays with refractive index sensing figures of merit approaching the theoretical limit. *Nat. Commun.* **4**, 2381 (2013).

18. Xue, T., Liang, W., Li, Y., Sun, Y., Xiang, Y., *et al.* Ultrasensitive detection of miRNA with an antimonene-based surface plasmon resonance sensor. *Nat. Commun.* **10**, 28 (2019).

19. Hui, X., Yang, C., Li, D., He, X., Huang, H., *et al.* Infrared Plasmonic Biosensor with Tetrahedral DNA Nanostructure as Carriers for Label-Free and Ultrasensitive Detection of miR-155. *Adv. Sci.* **8**, e2100583 (2021).

20. Yesilkoy, F., Arvelo, E. R., Jahani, Y., Liu, M., Tittl, A., *et al.* Ultrasensitive hyperspectral imaging and biodetection enabled by dielectric metasurfaces. *Nat. Photonics* **13**, 390–396 (2019).

21. Lee, I. H., Yoo, D., Avouris, P., Low, T.& Oh, S. H. Graphene acoustic plasmon resonator for ultrasensitive infrared spectroscopy. *Nat. Nanotechnol.* **14**, 313-319 (2019).

22. Liu, K., Huang, G., Li, X., Zhu, G., Du, W., *et al.* Vibrational Strong Coupling between Surface Phonon Polaritons and Organic Molecules via Single Quartz Micropillars. *Adv. Mater.* **34**, e2109088 (2022).

23. Wang, S., Cheng, Q., Lv, J.& Wang, J. Photonic crystal sensor based on Fano resonances for simultaneous detection of refractive index and temperature. *J. Appl. Phys.* **128**, (2020).
